# Formulation Development of Sublingual Cyclobenzaprine Tablets Empowered by Standardized and Physiologically Relevant Ex Vivo Permeation Studies

**DOI:** 10.3390/pharmaceutics13091409

**Published:** 2021-09-06

**Authors:** Haidara Majid, Andreas Puzik, Tanja Maier, Raphaela Merk, Anke Bartel, Hans-Christian Mueller, Bjoern B. Burckhardt

**Affiliations:** 1 Institute of Clinical Pharmacy and Pharmacotherapy, Heinrich Heine University, 40225 Dusseldorf, Germany; haidara.majid@hhu.de (H.M.); anke.bartel@hhu.de (A.B.); 2 Hexal AG, Analytical Development, 83607 Holzkirchen, Germany; andreas.puzik@novartis.com (A.P.); tanjam33@gmx.de (T.M.); merkraphaela@web.de (R.M.); hcm@dm-hc.org (H.-C.M.)

**Keywords:** sublingual drug administration, formulation development, cyclobenzaprine, patient-centered dosage form, oromucosal permeation, mucosal metabolism 201C

## Abstract

Suitable ex vivo models are required as predictive tools of oromucosal permeability between in vitro characterizations and in vivo studies in order to support the development of novel intraoral formulations. To counter a lack of clinical relevance and observed method heterogenicity, a standardized, controlled and physiologically relevant ex vivo permeation model was established. This model combined the Kerski diffusion cell, process automation, novel assays for tissue integrity and viability, and sensitive LC-MS/MS analysis. The study aimed to assess the effectiveness of the permeation model in the sublingual formulation development of cyclobenzaprine, a promising agent for the treatment of psychological disorders. A 4.68-fold enhancement was achieved through permeation model-led focused formulation development. Here, findings from the preformulation with regard to pH and microenvironment-modulating excipients proved supportive. Moreover, monitoring of drug metabolism during transmucosal permeation was incorporated into the model. In addition, it was feasible to assess the impact of dosage form alterations under stress conditions, with the detection of a 33.85% lower permeation due to salt disproportionation. Integrating the coherent processes of disintegration, dissolution, permeation, and metabolization within a physiological study design, the model enabled successful formulation development for cyclobenzaprine sublingual tablets and targeted development of patient-oriented drugs for the oral cavity.

## 1. Introduction

For certain special patient populations (e.g., children, the elderly, or patients with dysphagia, intestinal insufficiency, nausea, or trypanophobia), the common routes of drug administration (oral and parenteral) appear to be inappropriate and are often accompanied by poor adherence [1]. Administration via the oral mucosa as a patient- and indication-centered treatment offers a beneficial alternative. In addition to easier application, rapid and high systemic availability is achieved for the therapy of acute cases. Bypassing the digestive tract and first-pass metabolism allows for dose reduction [2], which facilitates patient safety and adherence by reducing the risk of side effects [3].

In order to support the progressive development and approval of oromucosal drugs, meaningful studies predicting pharmacokinetic properties are already essential at the preclinical stage [4]. During the preclinical stage, formulation development is a useful tool for influencing pharmacokinetic properties, with consideration given to the intended site of administration as well as the targeted patient population and indication. Decisive criteria include solubility, compatibility, stability, taste, and in particular drug release and absorption rate [5]. Conventionally, dissolution studies provide information on the drug release achieved, e.g., in quality control and stability studies, as well as on formulation development. However, in vitro/ex vivo permeation studies are useful for the investigation of the impact of the formulation on drug absorption, and allow for an extensive screening to guide formulation development and support transfer to in vivo studies [4]. Unlike dissolution studies, particularly for administration via the oral mucosa, drug permeation studies are not clearly regulated and the associated heterogeneity hinders their broad application. On the one hand, their use as continuous, decisive elements embedded into formulation development requires sensitivity and adaptation to physiological conditions in order that the pharmacokinetically/clinically relevant impacts of the formulations developed be detected. On the other hand, a standardized, comparable and regulatory implementable design with controlled processes is required to ensure efficient and reliable application [4,6,7,8]. These unmet requirements restrict the current application of ex vivo absorption studies to academic research and unregulated preliminary studies. For most other applications, elaborate, expensive, and ethically sensitive in vivo studies are the method of choice for the evaluation formulation of candidates [9,10]. In order to address this imbalance, an oromucosal ex vivo permeation model was successfully developed, standardized and validated [11]. Processes were automated and incorporated into a sophisticated control system which consisted of analytical quality controls and verification of tissue viability and integrity. Adapting the study design to physiological/clinical conditions allowed for excellent multiple correlations to sublingual in vivo data [12]. Moreover, the model was applied in comprehensive preformulation studies of oromucosal drug delivery [13]. Nonetheless, the expansion of the model from preformulation to formulation development for predicting the pharmacokinetically relevant impacts of developed dosage forms on absorption, especially under a physiology-based design and within clinically relevant application periods, has not yet been studied.

Cyclobenzaprine hydrochloride is a tricyclic dibenzocycloheptene muscle relaxant with a molecular weight of 311.8 g/mol, pK_a_ of 8.47, log P_OW_ of 5.2 and freely soluble in water. It is approved for the oral treatment of muscle pain and spasms with a daily dose of 15–30 mg [14]. Due to its antagonistic effects in the serotoninergic, histaminergic, and adrenergic systems, cyclobenzaprine is currently being investigated and discussed with regard to various additional indications, most notably for sleep disturbances in posttraumatic stress disorder (PTSD) and fibromyalgia [15,16]. PTSD is characterized by involuntary re-experiences and hyperarousal symptoms, for example sleep disturbances with nightmares, hypervigilance, and anxiety. The cross-national prevalence of PTSD in adults has been found to be 3.9% [17], and within this cohort 80–90% of the patients suffer from sleep disturbances [18]. Further potential applications for cyclobenzaprine include Alzheimer’s disease and long-COVID syndrome. In addition to these new potential indications, sublingual administration is also intended to reduce daytime side effects [19], such as somnolence, by providing a lower dose and avoiding the first-pass effect with the formation of the active and long-lived metabolite desmethyl cyclobenzaprine (norcyclobenzaprine) [20].

The aim of this study was to verify the power of the model to lead sublingual formulation development and thereby facilitate the targeted development of patient-centered oromucosal drugs. Moreover, an enhancement of oromucosal cyclobenzaprine permeation through optimized compositions was intended to exploit its therapeutic benefits and improve patient safety. Furthermore, the relevance of drug metabolism during transmucosal permeation was to be monitored and assessed, since data about metabolic activity in the oral cavity are limited. Finally, in this proof of concept, the sensitivity of the ex vivo permeation model for the purpose of the detection and classification of the impact of alteration on dosage forms was investigated.

## 2. Materials and Methods

### 2.1. Simultaneous Quantification of Cyclobenzaprine and Its Related Compounds

The simultaneous quantification of cyclobenzaprine hydrochloride (≥98%, Hetero drugs Ltd., Hyderabad, India), its main metabolite desmethyl cyclobenzaprine hydrochloride (99.8%, Toronto Research Chemicals, Toronto, Canada) and cyclobenzaprine N-oxide (96%, Toronto Research Chemicals, Toronto, Canada) as its major degradation product was performed by high-performance liquid chromatography coupled with electrospray ionization tandem mass spectrometry (LC-ESI-MS/MS) (Shimadzu Prominence, Shimadzu Europe, Duisburg, Germany; AB Sciex API 2000, Darmstadt, Germany). Chromatography was carried out on a Luna PFP (2) column (100.0 × 2.0 mm; 3 µm) with SecurityGuard PFP (2) pre-column (4.0 × 2.0 mm) (Phenomenex Ltd. Aschaffenburg, Germany) using cyclobenzaprine-d3 (98%, Sigma-Aldrich, Taufkirchen, Germany) as deuterated internal standard (IS). At a maintained column temperature of 55 °C, 0.1% formic acid (≥98%, p.a., Sigma-Aldrich, Taufkirchen, Germany) in water (LC-grade, Fisher Scientific, Schwerte, Germany) and 0.1% formic acid in acetonitrile (LC-grade, Fisher Scientific, Schwerte, Germany) served as mobile phases A and B at a flow rate of 450 µL/min. Gradient elution went from 7% to 72% of mobile phase B with a total run time of 5.3 min and an injection volume of 5 µL. The mass transitions and analyte specific parameters for detection in multiple reaction monitoring mode are summarized in Table 1. Mass spectrometric source parameters were set as follows: curtain gas (nitrogen): 20 psi, ion spray voltage: 2000 V, nebulizer gas (zero air): 42 psi, heater gas (zero air): 75 psi, collision gas (nitrogen): 7 psi and source temperature: 550 °C. Control of instrument and data acquisition were performed using Analyst^®^1.5.1 (AB Sciex, Darmstadt, Germany).

Validation of the simultaneous quantification method for cyclobenzaprine, desmethyl cyclobenzaprine and cyclobenzaprine N-oxide was performed according to international guidelines (EMA, FDA, and ICH Q2 guidelines [21,22,23]) for the parameters of linearity, accuracy, precision, sensitivity, dilution integrity, and recovery. In addition to method validation, each LC-MS/MS conducted run was monitored by system suitability tests, intra-run quality controls (QCs) and QCs regarding automated sample preparation. For this purpose, intra-run specifications were defined as a maximum relative error (RE) of ±15% (±20% at the lower limit of quantification (LLOQ)) and a correlation coefficient (r) of ≥0.995 for freshly prepared calibration curves.

### 2.2. Sublingual Formulation Development Guided by Permeation Studies

Preliminary preformulation studies [13] on the impact of pH and utilized excipients on transmucosal cyclobenzaprine permeability demonstrated a significant dependence on the addition of dipotassium hydrogen phosphate (dibasic phosphate) and also on environmental pH. These findings were transferred into formulation development through the manufacture of sublingual tablets (SLT) of three different compositions with varying amounts of dibasic phosphate (0.0 to 1.4%). All sublingual tablets were manufactured by direct compression using a rotary tablet press (Kilian RTS21, Romaco, Karlsruhe, Germany) and had a diameter and a weight of 0.6 cm and 76 mg, respectively. The ingredients of the sublingual tablets are compiled in Table 2.

With consideration given to optimized physiological and clinical conditions (e.g., low saliva volume of 150 µL for disintegration, short-term application due to indication and site of administration, sink conditions, etc.), the sensitivity of the model to variations in the sublingual formulation was investigated and compared with the outcomes from preformulation studies, as well as from dissolution studies as a conventional reference method (Section 2.3 and Section 2.4). The disintegration behavior of the developed sublingual tablets was visually assessed within a low-volume benchtop approach to mimic the physiological environment of the oral cavity. Therefore, 150 µL of fresh human saliva was added to the tablets and disintegration was monitored. The potential of the permeation model to lead formulation development was classified. Subsequently, the drug release, cumulative amount of permeated drug, steady-state flux and apparent permeability coefficient were assessed.

### 2.3. Standardized and Physiologically Relevant Permeation Model

#### 2.3.1. Model Set-up

An innovative, widely standardized and controlled ex vivo model, which has been described elsewhere [11], was used to study oromucosal permeability of cyclobenzaprine sublingual tablets (Section 2.2). The model consists of the combination of the following elements.

Fresh porcine esophageal mucosa, obtained by Naturverbund Thönes (Wachtendonk, Germany), separated and dermatomed to a thickness of 500 µm (Integra^®^ Dermal, Ratingen, Germany) was applied as a surrogate for oral mucosa [24,25,26,27]. The biological membrane was inserted in the Kerski diffusion cell and moistened with human saliva freshly collected under fasting conditions. After application of the formulation to be investigated, 100 µL of human saliva was pipetted on top of the sublingual tablet. The Kerski diffusion cell [28] allows for automated sampling with modified Hanson Research AutoPlus™ (Teledyne Hanson, Los Angeles, CA, USA), scheduled from 5 to 60 min after drug administration. In order to mimic physiological conditions, phosphate-buffered isotonic saline solution at pH 7.4 was used as an acceptor medium with environmental conditions of 37 °C temperature and 20% relative humidity (KBF 115 Constant Climate Chamber, Binder GmbH, Tuttlingen, Germany), and continuous stirring at 750 rpm (2mag Mixcontrol20, Munich, Germany) was maintained during the study period. The automation of sample preparation involved spiking cyclobenzaprine-d3 to the samples, dilution into the analytical calibration range, and agitation using an HTS PAL autosampler (CTC Analytics AG, Zwingen, Germany) and Chronos 5.0 software (Axel Semrau GmbH, Sprockhoevel, Germany). Coupling this with sensitive quantification by the validated LC-MS/MS method (Section 2.1.) made for a clinically representative study design (in terms of duration, measurement points, and therapeutic dose). Novel post-study tissue integrity and viability assays were incorporated to monitor and reevaluate the permeation results by excluding non-compliant measurements and diffusion cells, where applicable [11].

The cumulative amount of the permeated drug (Q_t_), the steady-state flux (J_SS_), and the apparent permeability coefficient (P_app_) were calculated using Equations (1)–(3) to assess permeability. Statistical differences were analyzed using an unpaired Student`s t-test with α = 0.05. The P_app_ values from varying amounts of dibasic phosphate of the sublingual tablets and those from the preformulation were correlated. The enhancement factor (EF) was used to rate the impact of formulation and excipient addition on cyclobenzaprine permeability (Equation (4)).

(1) $$Q_{t}=\frac{C_{n}\cdot V_{A}+(\sum_{n=1}^{n}C_{n-1})\cdot V_{R}}{A}\;[\mu g/\mathrm{cm}²]$$

Q_t_: Cumulative amount of permeated drug

C_n_: Drug concentration at time point n

C_n−1_: Drug concentration at previous time point

V_A_: Volume of acceptor chamber

V_R_: Removed volume

A: Available area for permeation

(2) $$J_{\mathrm{SS}}=\frac{{\Delta Q}_{t}}{\left(\Delta t\cdot A\right)}\;[\mu g/\mathrm{cm}²/h]$$

J_SS_: Steady-state flux

ΔQt: Difference in Qt between time points

Δt: Time difference

A: Available area for permeation

(3) $$P_{\mathrm{app}}=\frac{J_{\mathrm{SS}}}{\mathrm{CD}\;}\;[\mathrm{cm}\cdot s^{-1}]$$

P_app_: Apparent permeability coefficient

J_SS_: Steady-state flux

CD: Initial drug concentration

(4) $$\mathrm{EF}=\frac{P_{\mathrm{app}\;}\left(\mathrm{with}\;\mathrm{dibasic}\;\mathrm{phosphate}\right)}{P_{\mathrm{app}}\;\left(\mathrm{without}\;\mathrm{dibasic}\;\mathrm{phosphate}\right)}$$

EF: Enhancement factor

P_app_: Apparent permeability coefficient

#### 2.3.2. Metabolization of Cyclobenzaprine during Mucosal Permeation

The permeation model was extended by mucosal metabolic activity examination as an additional physiological model property. Therefore, the focus was on the formation of desmethyl cyclobenzaprine—the main active metabolite, which is responsible for clinically relevant daytime side effects—by mucosal administration. The cytochrome P450 isoenzymes 1A2, 3A4, and 2D6 are implicated in the catalysis of cyclobenzaprine demethylation [29,30]. To determine the extent of mucosal cyclobenzaprine metabolism, solutions containing 2.8 mg cyclobenzaprine and 1.1 mg dibasic phosphate were prepared. In this setup, esophageal mucosa, buccal (500 µm thickness) and sublingual mucosa (300 µm thickness) were examined to determine potential differences between metabolic activities of the esophagus and oral mucosa. Thus, permeation studies followed by extraction of the used mucosal membranes by 10 mL of methanol/water/formic acid (80:19:1 *v/v/v*) at 37 °C and 1000 rpm were conducted to detect the metabolized amount in the tissues. The relative mass balance of desmethyl cyclobenzaprine, as a relevant active metabolite, was calculated from the cumulative permeated amount, the membrane-extracted amount, and the amount in the applied donor solution. The different mucosa membranes were also incubated with solutions of 14 mg/mL cyclobenzaprine for 4 h at 37 °C to detect minor metabolite formation. As a negative control, served membranes were treated for at least 3 h with 1% formic acid in methanol to eliminate metabolic activity. In order to investigate metabolic activity in saliva, 2.8 mg cyclobenzaprine was added to fresh human saliva, incubated under the aforementioned conditions, and measured by LC-MS/MS.

Additionally, human liver microsomes (UltraPool™ HLM 150 Mixed Gender, Corning Inc., Amsterdam, The Netherlands) were used to study hepatic formation of desmethyl cyclobenzaprine, which is representative for first-pass metabolism. Microsomal metabolism studies were performed using a final concentration of 5 µM cyclobenzaprine at 37 °C. For this purpose, the substrate was added to an assay medium consisting of an NADPH regeneration system (NADPH Regenerating System Solution A and B, Corning Inc., Amsterdam, The Netherlands), 0.1 M potassium phosphate buffer and 0.25 mg human liver microsomes (HLM). Propranolol hydrochloride (100%, API, Caesar & Loretz GmbH, Hilden, Germany), verapamil hydrochloride (≥99, Sigma-Aldrich, Taufkirchen, Germany) and negative controls (drugs without human liver microsomes) served as assay controls. Samples were drawn at 0, 5, 15, 30, 45 and 60 min, according to sampling time points during the permeation experiments. The reaction was stopped by the addition of ice-cold acetonitrile.

#### 2.3.3. Impact of Alteration in Dosage Forms on Drug Liberation and Absorption

In this context, the permeation model was intended to detect dosage form alteration and assess its effect on drug absorption in order to estimate the implications on in vivo application. Therefore, the sublingual tablets were stored under stress conditions of 40 °C and 75% relative humidity for six months and subjected to the permeation model. Dissolution studies were conducted as a reference method (Section 2.4.). Sublingual tablets stored at ambient conditions of 25 °C with 60% relative humidity were used as a control.

In addition to dissolution and permeation behavior, surface analysis was performed using visual examination as well as light microscopy (Leica DM LM, Leica Microsystems, Heerbrugg, Switzerland) of the tablets and the aluminum–aluminum primary packaging material (Patz 38/ALU-H 20, Constantia Patz, Loipersbach, Austria). Further analysis and identification of residual compounds was performed by high-resolution time-of-flight mass spectrometry (TOF-MS) (AB Sciex TripleTOF 6600, Darmstadt, Germany), equipped with an IonDrive TurboV^®^ electrospray ionization source (AB Sciex, Darmstadt, Germany) in positive ion mode under the following conditions: curtain gas (nitrogen) at 25 psi, ion spray voltage at 5500 V, nebulizer gas (zero air) at 20 psi, heater gas (zero air) at 20 psi, source temperature at 100 °C, declustering potential at 30 V and collision energy at 10 V. The aluminum–aluminum primary packing materials foiled with Pentapack BP 540 (Kinrooi, Belgium) were rinsed with 2 mL tetrahydrofuran (≥99%, p.a., Sigma-Aldrich, Taufkirchen, Germany), evaporated under nitrogen stream at 40 °C with 300 rpm, and resuspended in methanol/water/formic acid (80:19:1 *v/v/v*).

### 2.4. Dissolution Studies

Dissolution studies, as a conventional pharmaceutical evaluation procedure in formulation development, were performed for the sublingual tablets to compare the power of dissolution studies versus the permeation model. Dissolution studies were conducted using baskets (USP apparatus 1) at 37 °C with a rotation speed of 50 rpm (Sotax AT7 smart, Sotax GmbH, Loerrach, Germany). The tablets were placed in 900 mL each of pH 6.8 phosphate buffer, and the released drug amount was quantified by LC-UV after sampling of 5 mL and filtering through 0.45 µm regenerated cellulose filter (Whatman GmbH, Dassel, Germany).

## 3. Results and Discussion

### 3.1. Simultaneous Quantification of Cyclobenzaprine and Its Related Compounds

**Figure 1:**
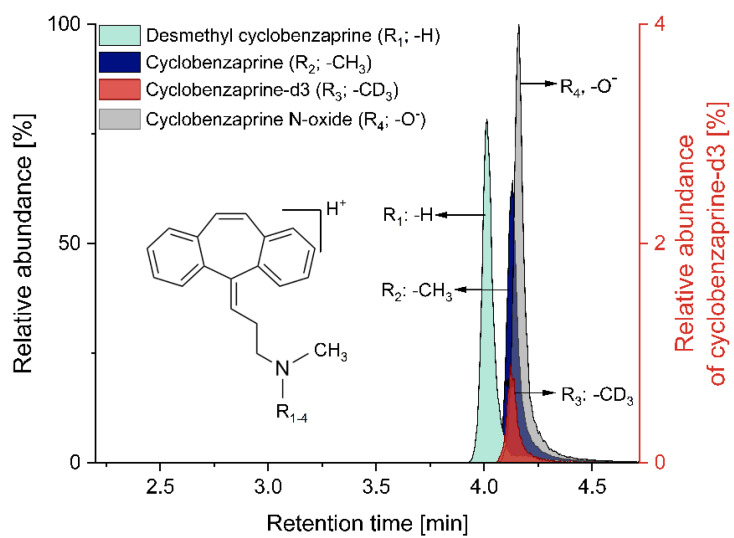
LC-MS/MS chromatogram of desmethyl cyclobenzaprine, cyclobenzaprine, cyclobenzaprine-d3 and cyclobenzaprine N-oxide with the respective structural formula.

A LC-MS/MS method for simultaneous quantification of cyclobenzaprine, desmethyl cyclobenzaprine and cyclobenzaprine N-oxide has been successfully validated. Figure 1 shows the chromatogram of the three analytes and the IS with the respective structural formula. Linearity of the method ranging from 0.93–952.38 ng/mL for each analyte was achieved by using 11 non-zero calibration levels. The best fit was revealed by quadratic regression (weighted 1/x^2^) with correlation coefficients (r) of ≥0.997.

The results for accuracy and precision (within-run and between-run) complied with the acceptance criteria of the international guidelines and are summarized in Table 3. Sensitivity was achieved by analyte responses at the LLOQ of ≥7 compared to zero standard and signal-to-noise ratios of ≥127:1. Dilution integrity (1:5, 1:10, 1:20) of cyclobenzaprine was confirmed using concentrations between 1500 and 12000 ng/mL with RE ranging from −6.16 to 14.31% and CVs of 0.82 to 3.75%. Automated sampling by modified Hanson Research AutoPlus™ was verified for all analytes, resulting in a RE of −13.50 to 11.24%.

Thus, a sensitive LC-MS/MS quantification method including automated sampling and sample preparation for cyclobenzaprine, desmethyl cyclobenzaprine, and cyclobenzaprine N-oxide was reported for the first time and used within the studies presented here.

### 3.2. Sublingual Formulation Development Guided by Permeation Studies

**Figure 2:**
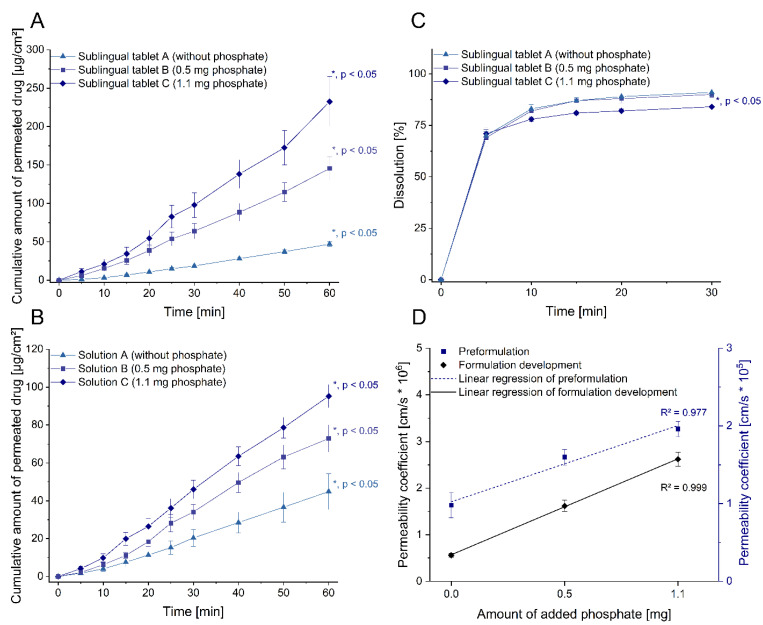
Impact of excipient addition in preformulation and formulation development of cyclobenzaprine. **A**: Cumulative amount of permeated drug per cm^2^ of the respective sublingual tablet (mean ± SEM; *n* ≥ 5). **B**: Cumulative amount of permeated drug per cm^2^ of the respective solution (mean ± SEM; *n* ≥ 5) adapted from [13], Int. J. Pharm. 2021. **C**: Dissolution of the respective sublingual tablet (mean ± SEM; *n* = 3). **D**: Correlation of obtained cyclobenzaprine permeability with the added amount of dibasic phosphate (mean ± SEM). *R*^2^: determination coefficient, SEM: standard error of the mean, *: significant value (p < 0.05; unpaired t-test).

In order to assess the usefulness of the permeation model in leading formulation development, the cyclobenzaprine permeation from the differently composed sublingual tablets was studied. In Figure 2A, the impacts of dibasic phosphate on cyclobenzaprine permeation using sublingual tablets are shown with calculated permeation lag times between 4.1 and 6.4 min. The cumulative amount of drug per area was improved significantly from 46.91 to 232.53 µg/cm² by increasing the amount of dibasic phosphate to 1.4% per tablet (EF of 2.89 and 4.68 for SLT-B and SLT-C, compared to SLT-A). Consequently, SLT-C improved cyclobenzaprine permeation most effectively. Increasing the permeation of cyclobenzaprine (pK_a_ of 8.47) by increasing pH values as a result of phosphate addition is in line with the pH-partition theory.

This trend is also consistent with results from preformulation studies (Figure 2B) using cyclobenzaprine solutions [13], in that a further increase in dibasic phosphate did not contribute to the improvement of permeation. According to a direct comparison of results from cyclobenzaprine solutions versus those from tablets, an absolute increase in permeation as well as in the EF (4.68 vs. 2.00) was superior for the tablets. This could be attributable to the different concentration gradients during disintegration of the tablets in a volume of 100 µL, compared to the drug solution which was normalized to the donor volume of 2 mL. In the studies presented here, the physiological conditions for permeation were predetermined, so the formulation had to both increase and maintain pH in the microenvironment by its excipients to achieve the predicted improvement in permeation. Therefore, the permeation profiles of solution A and sublingual tablet A without excipient addition were comparable. Due to the resulting pH in solution A as well as after administration of SLT-A, cyclobenzaprine was present almost completely ionized. This indicates that the paracellular pathway is the most likely for diffusion. As its capacity is limited, a less sensitive response to concentration changes can be expected [31].

Moreover, based on the previous study of solutions consisting only of the two components cyclobenzaprine and varying proportions of dibasic phosphate, the permeation-enhancing effect can be attributed to the addition of dibasic phosphate acting by controlling the pH at the site of administration. Accordingly, only the phosphate portion was changed in the sublingual tablet compositions to selectively determine its effect on drug permeation. Analogously, the addition of dibasic phosphate to the tablets increased the permeability of cyclobenzaprine, which highlighted the effect of phosphate on permeability, while permeation between the sublingual tablet A and solution A (both without dibasic phosphate) was comparable. Thus, the relationship between the obtained permeability coefficients and the amount of dibasic phosphate added showed a linear correlation in the preformulation study (coefficient of determination R² of 0.977) as well as for the manufactured sublingual tablets (R² of 0.999).

**Figure A1:**
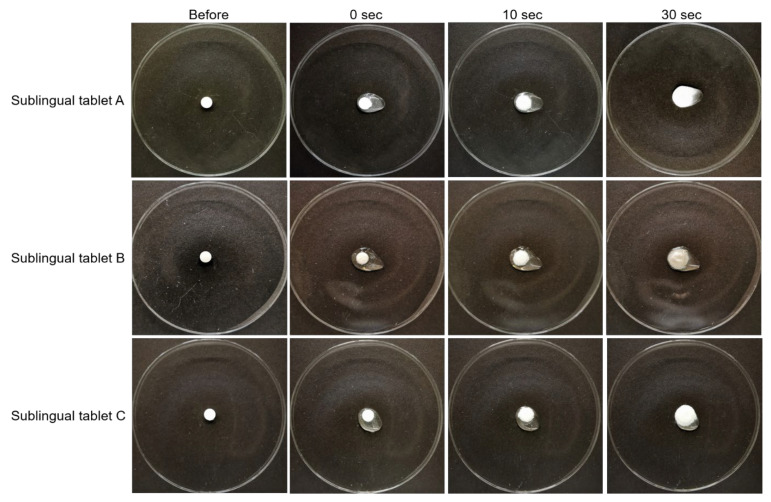
Disintegration of cyclobenzaprine sublingual tablets after addition of 150 µL freshly collected human saliva in dependence on time.

Dissolution studies for evaluation of the new formulations showed no significant difference in the profiles of SLT-A and SLT-B, with 91% and 90% drug release, respectively (Figure 2C). In contrast, a significantly lower drug release was measured with SLT-C (84%). Thus, an inconsistent rank order was observed compared to the preformulation and formulation development. Despite the use of a phosphate buffer medium, dissolution studies were not able to discriminate between the effects of formulation ingredients. For ionizable drugs such as cyclobenzaprine, the preferred properties for solubility or release are partially opposite to those for permeability, limiting the exclusive use of dissolution to assess the developed oromucosal formulations regarding absorption-affecting parameters and underlining the requirement for standardized permeation studies. During the permeation studies, a rapid disintegration of the tablets was observed, which was further investigated in a benchtop approach. The visually detected disintegration time of SLT-A, SLT-B and SLT-C after addition of saliva was uniform within 30 s. The records of the time course of the disintegration are shown in Figure A1. The oromucosal model presented combines absorption under physiological conditions, taking parallel processes such as disintegration, dissolution and permeation into consideration. In addition, it provides information on the amount of drug at the application site, as well as its absorption capacity, so that technological outcomes can be linked to clinical significance.

In summary, the sublingual tablets were successfully evaluated in terms of resulting permeation as part of the formulation development for cyclobenzaprine. Furthermore, transferability between preformulation and the final dosage forms allows, in contrast to dissolution, the screening and grading of compositions and specific additives even before the dosage forms are manufactured. Under physiological conditions and standardized procedures, the selection of the final composition (SLT-C) was feasible and enables the targeted transfer into the following in vivo processes. As a result, a reliable and representative screening of the formulation candidates supports their development and optimization, and enables a reduction in the number of animal experiments required as well as a reduction in the resource expenditure associated with such experiments.

### 3.3. Metabolism of Cyclobenzaprine during Mucosal Administration

**Figure 3:**
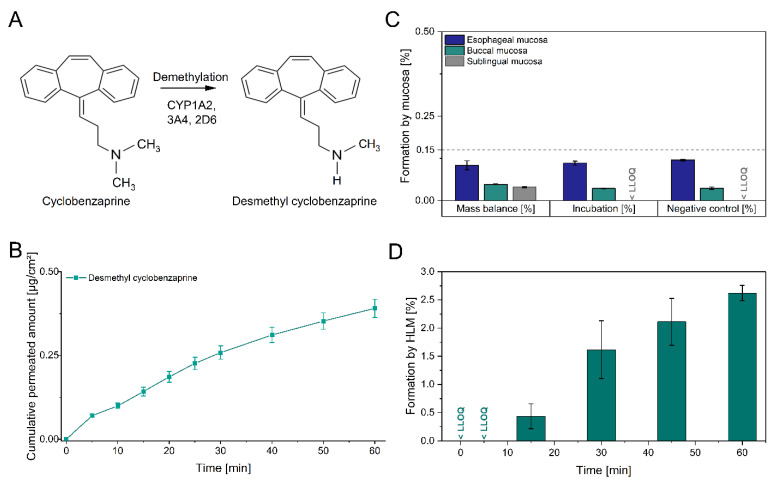
Cytochrome P450 metabolism of cyclobenzaprine. **A**: Scheme of cyclobenzaprine demethylation by CYP isoenzymes. **B**: Cumulative amount of permeated desmethyl cyclobenzaprine per cm^2^ (mean ± SEM; *n* = 8). **C**: Formation of desmethyl cyclobenzaprine by different mucosae and approaches (mean ± SEM; *n* ≥ 2). **D**: Formation of desmethyl cyclobenzaprine by human liver microsomes per time (mean ± SEM; *n* = 3). *HLM: human liver microsomes, LLOQ: lower limit of quantification*.

In order to monitor the drug metabolism during transmucosal permeation, the formation of desmethyl cyclobenzaprine was analyzed. A remarkably low cumulative amount of 0.39 µg/cm² was determined for desmethyl cyclobenzaprine, compared to a cyclobenzaprine permeation of 95.23 µg/cm² after 60 min (Figure 3B). The relative mass balance of desmethyl cyclobenzaprine resulted in low values (from 0.04 to 0.11%) for the esophageal, buccal, and sublingual mucosa (Figure 3C). Concentrations of desmethyl cyclobenzaprine around and below the LLOQ were measured even when incubated with high cyclobenzaprine solutions of 14 mg/mL. In conclusion, no substantial formation by the mucosal tissues was detected as comparable amounts of desmethyl cyclobenzaprine were also found in the negative controls.

Thus, the overall percentages of less than 0.15% in each metabolization approach were in line with the degree of desmethyl cyclobenzaprine impurity and might be derived from drug synthesis. The U.S. Pharmacopoeia defines desmethyl cyclobenzaprine as a compound-related impurity B with an acceptable level of ≤0.15% [32]. In contrast to mucosal tissue, HLM studies demonstrated continuous formation of desmethyl cyclobenzaprine of up to 2.5% relative to the applied amount of cyclobenzaprine (Figure 3D). In the negative control, the amount of desmethyl cyclobenzaprine was below the LLOQ. Intrinsic clearance of propranolol and verapamil as assay controls was consistent with reported data. In the literature, cyclobenzaprine is defined as an extensively metabolized drug with enterohepatic circulation [33]. The ratio of urinary desmethyl cyclobenzaprine to cyclobenzaprine was at least fivefold lower in clinical trials when administered intravenously, therefore bypassing the first-pass effect, compared to oral administration [34]. A decreased formation of desmethyl cyclobenzaprine is linked to a reduction in daytime side effects. Since no mucosal formation of desmethyl cyclobenzaprine was detected by the oral mucosa and this route of administration circumvents the first-pass effect as well, sublingual delivery seems to compare favorably with approved oral administration. 

### 3.4. Impact of Alteration in Dosage Forms on Drug Liberation and Absorption

**Figure 4:**
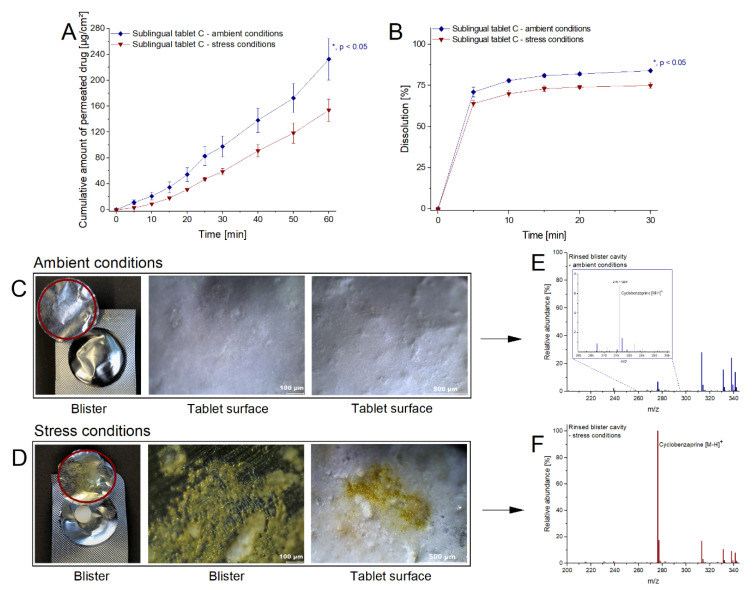
Alteration of cyclobenzaprine sublingual tablets under ambient and stress conditions. (**A**): Cumulative amount of permeated drug per cm^2^ of the respective sublingual tablet stored (mean ± SEM; *n* ≥ 4). (**B**): Dissolution of the respective sublingual tablet stored (mean ± SEM; *n* = 3). (**C**,**D**): Visual and microscopic inspection of the primary packaging material and the tablet surface after storage under ambient conditions and stress conditions, respectively. (**E**,**F**): TOF-MS scan of the rinsed residuals from packaging material after storage under ambient conditions and stress conditions, respectively. *m/z: mass-to-charge ratio, SEM: standard error of the mean*, *: significant value (p < 0.05; unpaired t-test).

Within formulation development, SLT-C proved to be the most promising candidate and was chosen as the final composition. Accordingly, stability studies of the sublingual tablets under stress conditions (40 °C and 75% relative humidity for six months) were conducted in order to detect and compare formulation-related alterations using dissolution studies and the permeation model, while also estimating their clinical impact. Figure 4A,B illustrate the influence of stress storage conditions on dissolution and permeation. The cumulative permeated amount decreased significantly by 33.85% while dissolution decreased by 10.71% (based on the respective last measurement time) compared with storage under ambient conditions. In addition to the measurable decrease in release and permeation, a yellow oily coloration was observed in the aluminum–aluminum primary packaging material of the stressed sublingual tablets (Figure 4C,D), which was not visible after ambient storage. Light microscopy images of the sublingual tablets showed a uniform flat surface after ambient storage; however, under stress conditions the surface appeared much more porous with yellowish crystals on the tablet surface as well as in more pronounced form in the primary packaging material. TOF-MS spectra of the rinsed primary packaging material showed an approximately tenfold higher intensity for cyclobenzaprine under stress compared to ambient conditions, and no substantial signals for cyclobenzaprine N-oxide—which is described as the main degradation product of oxidation [35]—for either storage (Figure 4E,F).

Liu et al. reported a total of 15 degradation products for cyclobenzaprine based on three degradation pathways (exocyclic, endocyclic as well as oxidation of the tertiary amino group) by forced degradation studies [35]. None of the reported degradation products were detected in our studies. Salt disproportionation, rather than degradation, provided an explanation for the significantly higher cyclobenzaprine signal in the stressed primary packing material of the final composition SLT-C. Salt disproportionation is a process where the microenvironmental pH exceeds the pH of the maximum solubility of a basic drug and results in the conversion of the salt to the free base [36]. In solid dosage forms, salt disproportionation is both solution- and excipient-mediated. The increased humidity of 75% and the use of hygroscopic excipients (crospovidone) may have led to the initial moistening of the tablet surface with the formation of an aqueous diffusion layer and its internal migration [37], resulting in a basic microenvironmental pH due to the use of dibasic phosphate. This may have exceeded the pH of maximum solubility of cyclobenzaprine as a weak base with a pK_a_ of 8.47 and gradually induced the thermodynamically driven crystallization and accumulation of the lipophilic compound (log P_OW_ = 5.2) on the primary packaging material through the tablet surface. Further factors affecting the extent of disproportionation are the ratio of API to basic excipient, the temperature (40 °C rather than 25 °C), and the amorphization of compressed tablets [38].

The different results in dissolution and permeation according to alteration of the dosage forms underline the effectiveness of the permeation model from Section 3.2, whereas the discrepancy between the effects can be explained as follows. The sole adoption of dissolution studies for sublingual tablets does not consider the physiological situation, hence the storage effects are only defined by the drug loss to the primary packaging material, which was not accessible for dissolution. In addition, the precipitated drug amount on the tablet surface might be resolved due to the pH of 6.8 in dissolution studies. During the disproportionation reaction, the dibasic phosphate dissolved in the aqueous layer and contributed to the assumed alkalization of the medium, which caused its migration out of the tablet. In Section 3.2, the substantial influence of reduced phosphate amount on the permeation capacity of cyclobenzaprine was presented. In addition to the steps of disintegration and dissolution, the model also considers the drug permeation interaction in physiological approximation instead of using the artificial vessel approach. Thus, the influence of the tablet texture, the reduced phosphate content, the solubility of the precipitated drug, the concentration gradient, the composition, and the available volume of the biological medium on the multiple processes were all considered, whereas these influences are suppressed in the dissolution approach. Therefore, a loss measurement of 10.71% in dissolution might underestimate the actual clinical impact of lower drug exposure. Using the permeation model enabled a clinically representative description of the potential impact on patients. This emphasizes the suitability and sensitivity of the physiologically relevant permeation model for the detection and classification of alterations and the instability of solid oromucosal dosage forms, and thus also for estimation of their clinical relevance.

Within this proof of concept approach, the performance of the model in several stages of formulation development was demonstrated by the clinically representative assessment of optimizations in the sublingual dosage form. The permeation of cyclobenzaprine was thereby enhanced by a factor of 4.68 in the final composition, enabling dose reduction and consequently contributing to patient safety. The metabolization of cyclobenzaprine to desmethyl cyclobenzaprine during mucosal permeation was successfully integrated into the permeation model. It has been shown that no desmethyl cyclobenzaprine was formed by the permeation of various mucosal membranes, thus supporting the improvement of patient adherence by reducing side effects associated with the active metabolite [20].

In addition to previously reported model suitability in preformulation and in vivo predictivity, an advancement to sublingual formulation development was achieved within this study. The parallel processes of disintegration, dissolution, permeation and metabolization were integrated in a physiological study design and a standardized controlled environment, allowing efficient and targeted drug development. In comparison with conventional dissolution studies, the model obtained more sensitive and distinct outcomes by its adaptation to sublingual administration, with regard to the optimization of composition in formulation development as well as the impact of dosage form stability. Therefore, the model proves its usefulness as a bridging element between conventional in vitro characterization and pharmacokinetic in vivo studies. Oromucosal administration contributes to patient adherence through broad patient acceptance, ease of administration, and therapeutic safety [39] and also allows for patient- and symptom-tailored drug delivery. However, for the evaluation of patient-oriented dosage forms in early development stages, appropriate ex vivo studies are limited. For these development-intensive formulations in particular, the model presented here enables a reliable and physiologically/clinically relevant screening mechanism to support the advancement of patient-oriented drugs under resource-efficient conditions.

## 4. Conclusions

A standardized and physiologically relevant ex vivo model on oromucosal permeability was successfully applied in order to lead the sublingual formulation development of cyclobenzaprine, with more than fourfold enhancement in permeation achieved by optimizing the formulation. Advanced optimization of the model facilitated the decisive assessment of oromucosal formulations combining the simultaneous impact on disintegration, dissolution, permeation and metabolization. In addition, the suitability of the method of detection and evaluation of dosage form alteration and its impact on drug absorption was demonstrated. The effectiveness and predictivity of the presented model thus enable its application for the purposive development of patient-centered intraoral dosage forms.

## Figures and Tables

**Table 1:** Mass spectrometric conditions for cyclobenzaprine, desmethyl cyclobenzaprine and cyclobenzaprine N-oxide. *ESI: electrospray ionization, m/z: mass-to-charge ratio, msec: millisecond.*

Analyte-Specific Parameters	Cyclobenzaprine	Cyclobenzaprine N-Oxide	Desmethyl Cyclobenzaprine	Cyclobenzaprine-d3
Mass transition [m/z]	276.2 → 215.0	292.4 → 231.2	262.4 → 231.2	279.2 → 215.0
Declustering potential	55 V	55 V	55 V	55 V
Focusing potential	380 V	380 V	380 V	380 V
Entrance potential	10 V	9 V	9 V	10 V
Cell entrance potential	21 V	10 V	10 V	21 V
Collision energy	61 V	25 V	25 V	61 V
Cell exit potential	10 V	10 V	10 V	10 V
Mode	ESI (+)			
Dwell time	80 msec			

**Table 2:** Compositions of sublingual tablets. API, Active pharmaceutical ingredient; SLT, Sublingual tablet.

Ingredients	Amount [%]	Amount [mg]
Cyclobenzaprine hydrochloride (API) (Hetero drugs Ltd., Hyderabad, India)	3.7	2.80
Crospovidone (Kollidon CL, BASF, Ludwigshafen, Germany)	5.3	4.00
Peppermint aroma (Symrise, Holzminden, Germany)	3.7	2.80
Sodium stearyl fumarate (Pruv, JRS Pharma, Rosenberg, Germany)	2.6	2.00
Dipotassium hydrogen phosphate (Merck KGaA, Darmstadt, Germany)	0.0 (SLT-A); 0.7 (SLT-B); 1.4 (SLT-C)	0.00 (SLT-A); 0.53 (SLT-B); 1.05 (SLT-C)
Silicon dioxide (Syloid 244 FP, Grace, Worms, Germany)	1.3	1.00
Sucralose (Merck KGaA, Darmstadt, Germany)	0.3	0.25
Levomenthol (L-Menthol, BASF, Ludwigshafen, Germany)	0.03	0.02
Mannitol (Pearlitol 100 SD, Frankfurt, Germany)	ad 100	ad 76.00
**Physical attributes**		
Shape	White round sublingual tablet	
Diameter	0.6 cm	
Height	0.27 cm	
Weight	76.0 mg	

**Table 3:** Summary of accuracy and precision results for cyclobenzaprine, desmethyl cyclobenzaprine and cyclobenzaprine N-oxide (accuracy presented as mean relative error and precision as coefficient of variation, *n* = 5 per run). *CV, coefficient of variation; LLOQ, Lower limit of quantification; QC, quality control.*

Analyte	Quality Control [ng/mL]		Relative Error [%]				CV [%]	
			Within-Run			Between-Run	Within-Run	Between-Run
			Run 1	Run 2	Run 3			
Cyclobenzaprine	QC high	476.19	+4.50	−0.24	+1.97	+2.08	3.46	3.87
	QC middle	59.52	+1.04	+3.27	+3.71	+2.67	2.73	2.81
	QC low	3.72	−6.06	−6.74	−5.03	−5.94	4.08	4.08
	QC LLOQ	0.93	+13.33	+8.68	+16.63	+12.82	4.28	5.21
Desmethyl cyclobenzaprine	QC high	476.19	+4.15	−3.98	−5.18	−0.66	3.57	6.07
	QC middle	59.52	+1.30	+1.95	+1.76	+2.27	2.25	2.25
	QC low	3.72	−2.21	−5.04	−4.70	−3.41	4.25	4.25
	QC LLOQ	0.93	+1.73	+8.19	+0.35	+3.99	3.54	5.14
Cyclobenzaprine N-oxide	QC high	476.19	−0.74	−8.36	−2.30	−3.21	3.77	5.37
	QC middle	59.52	−5.49	−3.61	−1.34	−2.91	2.20	2.92
	QC low	3.72	−0.47	−3.99	+0.57	−0.71	4.92	5.02
	QC LLOQ	0.93	+0.25	−2.41	−3.18	−1.24	2.83	3.13

## Data Availability

The data supporting the findings of this study can be provided upon request to the corresponding author, Burckhardt, B.B., if applicable.
